# Changes in Buprenorphine and Methadone Supplies in the US During the COVID-19 Pandemic

**DOI:** 10.1001/jamanetworkopen.2022.23708

**Published:** 2022-07-26

**Authors:** Annie Y. Chen, David Powell, Bradley D. Stein

**Affiliations:** 1 RAND Corporation, Boston, Massachusetts; 2 Pardee RAND Graduate School, Santa Monica, California; 3 RAND Corporation, Washington, DC; 4 RAND Corporation, Pittsburgh, Pennsylvania

## Abstract

**Importance:**

The opioid crisis has been exacerbated by the COVID-19 pandemic in the US, with concerns over major disruptions to medication treatment of opioid use disorder.

**Objective:**

To investigate whether the COVID-19 pandemic was associated with disruption of buprenorphine and methadone supplies in the US.

**Design, Setting, and Participants:**

This repeated cross-sectional study used ARCOS (Automated Reports and Consolidated Ordering System) data, which monitor the flow of controlled substances in the US, from January 1, 2012, through June 30, 2021. Manufacturers and point of sale or distribution at the dispensing or retail level, including hospitals, retail pharmacies, clinicians, midlevel clinicians, and teaching institutions, were included in the analysis.

**Exposures:**

COVID-19 pandemic.

**Main Outcomes and Measures:**

Quarterly supplies of buprenorphine and methadone per capita in milligrams.

**Results:**

The per capita supply of methadone dropped from 13.2 mg in the first quarter of 2020 to 10.5 mg in the second quarter of 2020, whereas the per capita supply of buprenorphine increased from 3.6 mg to 3.7 mg in the same period. The per capita supply of methadone declined 20% (−2.7 mg) in the second quarter of 2020 compared with the first quarter of 2020, and the supply had not returned to 2019 levels as of June 2021, whereas the supply of buprenorphine per person increased consistently during the same period. There were considerable state disparities in the reduction of the methadone supply during the pandemic, with many states experiencing pronounced per capita supply decreases, including reductions as great as 50% in New Hampshire and Florida. These decreases in per capita methadone supply were not compensated by proportional increases in the per capita buprenorphine supply (linear fit, 0.17 [95% CI, −0.43 to 0.76]; *P* = .47).

**Conclusions and Relevance:**

This cross-sectional study of buprenorphine and methadone supplies during the COVID-19 pandemic found a pronounced decline in the methadone supply but no disruption to the buprenorphine supply. Future research is needed to explain the pronounced state disparities in the methadone supply.

## Key Points

**Question:**

Is the COVID-19 pandemic associated with changes in the US supplies of buprenorphine and methadone?

**Findings:**

This cross-sectional study used quarterly state data on methadone and buprenorphine supplies and found that the per capita supply of methadone declined significantly in the second quarter of 2020 and had not returned to 2019 levels as of June 2021. The per capita supply of buprenorphine increased during the same period.

**Meaning:**

These findings suggest that during the COVID-19 pandemic, the supply of methadone was disrupted, while the supply of buprenorphine was not.

## Teaser

This cross-sectional study examines disruptions to the buprenorphine and methadone supplies to treat opioid use disorder during the COVID-19 pandemic in the US.

## Introduction

The COVID-19 pandemic has exacerbated the opioid crisis in the US.^1^ Individuals with opioid use disorder (OUD) are at heightened risk of opioid misuse during the pandemic due to increased stress and social isolation.^2^ The number of opioid overdose deaths in 2020 was the highest ever recorded in a single year.^3^ Preliminary data from the Centers for Disease Control and Prevention show that deaths due to opioid overdose have been increasing since the spring of 2020, when stay-at-home orders to contain the COVID-19 outbreak were implemented around the country.^3,4^ As the dual crisis^1^ proceeds with substantial growth in fatal and nonfatal overdoses,^4,5^ access to effective treatment for OUD is critical.

One major area of concern has been the availability of medication for OUD (MOUD), with medications such as buprenorphine and methadone being the most effective treatments available.^6,7^ Past research has shown that even short delays in access may adversely affect MOUD initiation, illicit opioid use, and overdose death.^8,9,10^ In light of the potential adverse effects of MOUD disruption, the Substance Abuse and Mental Health Service Administration and states relaxed the restrictions related to MOUD, facilitating greater use of telehealth and greater flexibility in the provision of take-home methadone doses starting in March 2020.^11,12^

Nevertheless, methadone for OUD can still only be dispensed by opioid treatment programs (OTPs), in contrast to patients who can be prescribed buprenorphine by any clinician with a waiver to prescribe it, which may lead to greater disruption on the supply of methadone, especially for patients initiating MOUD. Furthermore, clinicians with waivers may prescribe buprenorphine to new patients for treatment without first conducting an in-person evaluation, whereas OTPs can only admit new patients and initiate treatment with methadone if an initial in-person physical evaluation is performed.^13^ Telehealth requirements also vary. For existing patients, dispensing of methadone by OTPs requires audiovisual telehealth visits, whereas early in the pandemic, OTPs received approval to provide buprenorphine through audio-only visits.^14^ Finally, buprenorphine is routinely provided through retail pharmacies, but methadone for OUD is typically dispensed only through OTPs,^15^ where the increased need for social distancing and isolation might cause disruption of care.^16,17,18^

Literature on access to MOUD during the COVID-19 pandemic has expanded, but studies characterizing national-level changes are still limited. One study using national prescription data^19^ showed that existing patients were able to maintain access to buprenorphine for OUD throughout the pandemic, but the number of new patients receiving buprenorphine for OUD decreased by approximately 25% at the beginning of the pandemic and later recovered. Another study using a nationwide sample of privately insured individuals^20^ found that the rate of use of buprenorphine remained stable during the early stages of the pandemic. Finally, researchers examining claims for commercial and Medicare Advantage enrollees in the first 3 months of the pandemic found no decrease in medication fills or clinician visits among patients already receiving MOUD; however, fewer individuals initiated MOUD.^21^

To our knowledge, the Automated Reports and Consolidated Ordering System (ARCOS), a commonly cited data source for characterizing national MOUD availability,^15,22^ is the only database tracking the commercial distribution of controlled substances in the US that has released data through the first half of 2021. Using ARCOS, we examined buprenorphine and methadone supplies during the pandemic. We hypothesized that the supply of methadone would exhibit more disruption than that of buprenorphine and that owing to fewer barriers to initiate buprenorphine treatment for new patients during the pandemic, states with a greater decrease in the supply of methadone would experience a parallel increase in the supply of buprenorphine.

## Methods

### Study Overview and Data Sources

This repeated cross-sectional study used ARCOS annual published retail drug summary data from January 1, 2012, through December 31, 2020, and the 6-month report for January 1 through June 30, 2021, to examine buprenorphine and methadone supplies before and during the COVID-19 pandemic.^23^ ARCOS is a national data collection system in which manufacturers and distributors report controlled substance transactions to the Drug Enforcement Administration. Reported details include manufacturers, inventory, point of sale, and distributions of substances at the dispenser or retail level (ie, hospitals, retail pharmacies, clinicians, midlevel clinicians, and teaching institutions) but contain no information on who received the medication or why these substances were dispensed (eg, MOUD, pain control). We used quarterly data of drug distribution by state and annual data of drug distribution by type of registrant in the annual summary data.^23^ We followed the Strengthening the Reporting of Observational Studies in Epidemiology (STROBE) reporting guideline for cross-sectional studies. The RAND Institutional Review Board determined that the study was exempt from review because it did not involve human participants.

We used quarterly population data from the US Census Bureau to calculate buprenorphine and methadone supplies in milligrams per capita and plotted these trends over time.^24^ A standard buprenorphine dose is smaller in terms of milligrams than a standard methadone dose^25^; therefore, we focused on the respective trends over time but did not compare the mean per capita supplies of buprenorphine and methadone directly. We also examined differences at the state level, given that before the COVID-19 pandemic, there were significant state differences in distribution of methadone for OTPs.^26^ All metrics of supply at the state level were scaled by population size using data from the US Census Bureau.^27^ To show state-level disparities in methadone supply changes, we mapped the percentage change in per capita methadone supply in quarter 3 of 2020 through quarter 2 of 2021, the most recently available 4 quarters of data, compared with quarters 1 through 4 of 2019. Both periods encompass 4 consecutive quarters, accounting for any common seasonal effects.

### Statistical Analysis

We compared state-level growth in the buprenorphine supply with state-level changes in the methadone supply and fit a linear association between these 2 variables. Using the total supply in 2019 in each state as a benchmark, we calculated the percentage change in supplies of methadone and buprenorphine in quarter 3 of 2020 through quarter 2 of 2021 relative to quarters 1 through 4 of 2019. This analysis provides evidence about whether states incurring the largest reductions in methadone supply experienced compensating increases in buprenorphine supply or, alternatively, experienced worse growth in buprenorphine supply. We report *P* values and 95% CIs to show whether a linear association exists between state-level changes in buprenorphine and methadone supplies. Two-sided *P* < .05 was considered statistically significant. All analyses were conducted using Stata/MP, version 16.1 (StataCorp LLC).

## Results

**Figure 1.**
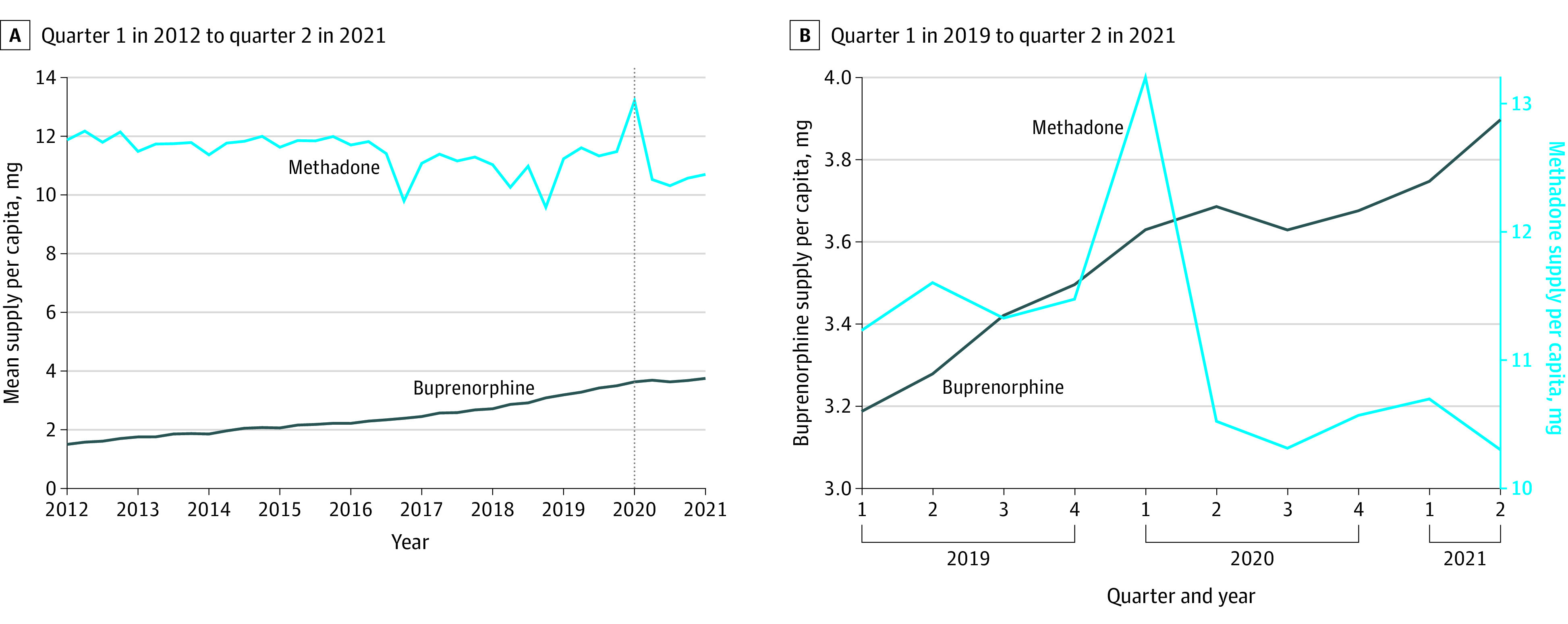
Mean Per Capita Supply of Buprenorphine and Methadone During the Study Period Data are from ARCOS (Automated Reports and Consolidated Ordering System). The vertical dashed line indicates the outbreak of COVID-19 in the US.

The mean per capita supply of buprenorphine rose steadily from 1.5 mg in quarter 1 of 2012 to 3.9 mg in quarter 2 of 2021 (Figure 1). Buprenorphine was predominantly dispensed in pharmacies, with little variation in the last decade (eFigure 1 in the Supplement). During the pandemic, the supply of buprenorphine was stable with the per capita supply increasing slightly from 3.6 mg in quarter 1 of 2020 to 3.7 mg in quarter 2 of 2020. By June 2021, the mean per capita supply had grown 7% compared with quarter 1 of 2020.

In contrast, the per capita methadone supply had greater fluctuations, and the proportion of methadone dispensed in OTPs had increased from 60% in 2012 to more than 88% in 2020 (eFigure 2 in the Supplement). There were substantial single-quarter dips in quarter 4 of 2016, quarter 4 of 2018, and quarter 2 of 2020 (Figure 1). The transitory dips in 2016 and 2018, which were 14% and 13% in magnitude, respectively, compared with the previous quarter, recovered in the next 2 quarters. On the contrary, the reduction in the mean supply of methadone per capita from quarter 1 to quarter 2 in 2020 was the biggest in recent years, dropping 20% from 13.2 mg in quarter 1 to 10.5 mg in quarter 2 (Figure 1). Part of this large decline might be attributable to an increase in methadone supply in quarter 1 of 2020, but that increase did not appear to explain the full decline that persisted for 1 year. Compared with the mean methadone supply per capita in 2019 (11.4 mg), the decline in quarter 2 of 2020 was still substantial (−8%) and the supply had not recovered to its 2019 level, even by June 2021 (Figure 1). The persistence of this decline is unique relative to historical methadone supply trends (Figure 1).

**Figure 2.**
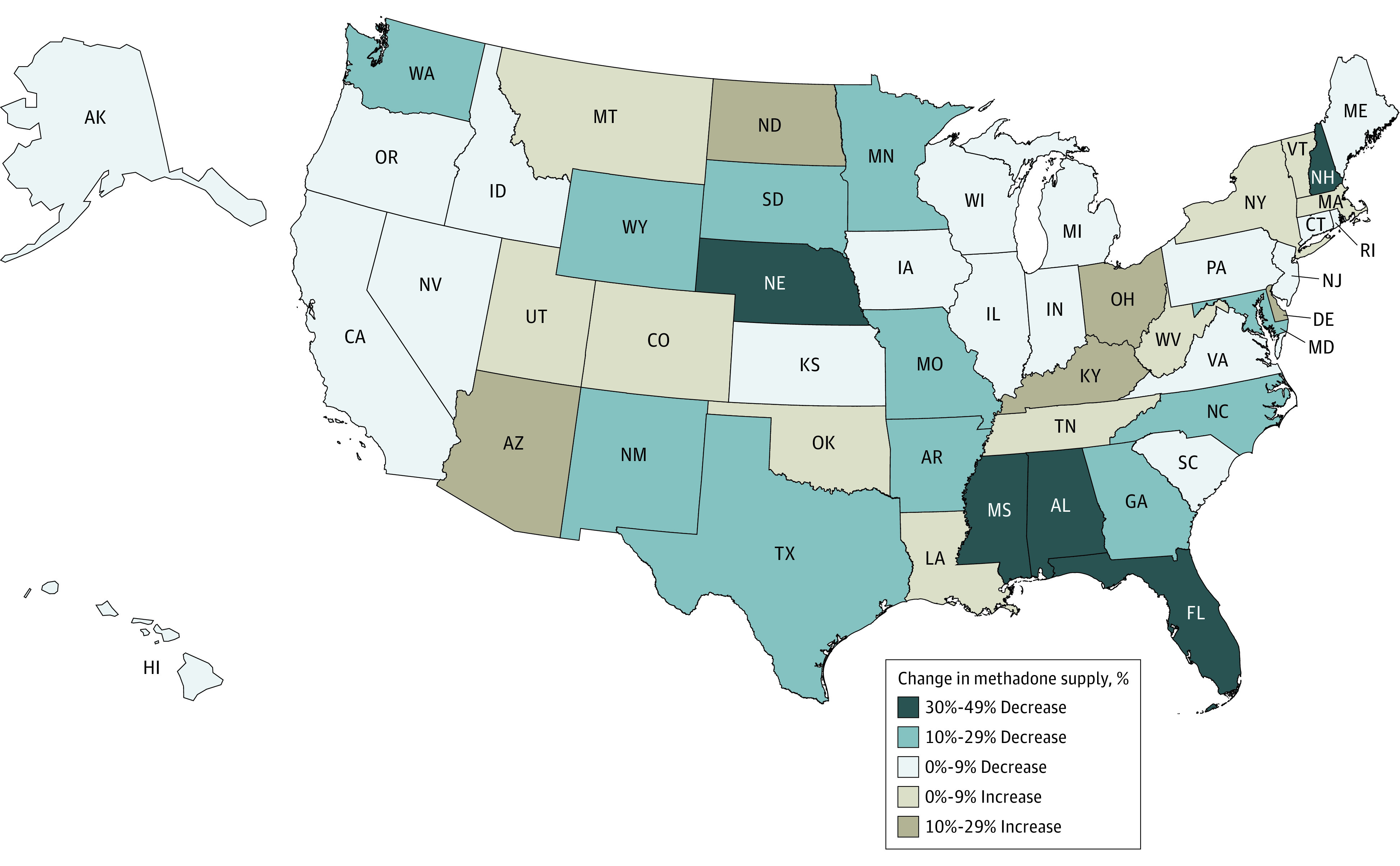
Percentage Change in Methadone Supply by State Data are represented for quarter 3 (Q3) of 2020 to Q2 of 2021 and Q1 to Q4 of 2019. Results were calculated using ARCOS (Automated Reports and Consolidated Ordering System) data and state population estimates from the US Census Bureau.^27^

Although we observed notable growths or, in rare cases, no change in the supply of buprenorphine across states, there was substantial geographic variation in the changes in methadone supply (Figure 2). Compared with 2019 per capita levels (11.4 mg), the per capita supply of methadone in quarter 3 of 2020 through quarter 2 of 2021 decreased by 0.9 mg nationally, and supply reductions were observed in 35 states and Washington, DC. New Hampshire and Florida both experienced nearly 50% decreases in the per capita supply of methadone, whereas the supply in Alabama, Mississippi, and Nebraska during the pandemic dropped more than 30% relative to the supply per capita in 2019. In total, 16 states experienced more than a 10% decrease in methadone supply. Many of the states with the largest decreases in the per capita methadone supply (eg, Florida, Mississippi, and Alabama) were in the South. In contrast, 15 states experienced increases in the per capita methadone supply; for example, Ohio’s per capita methadone supply increased by 26%.

**Figure 3.**
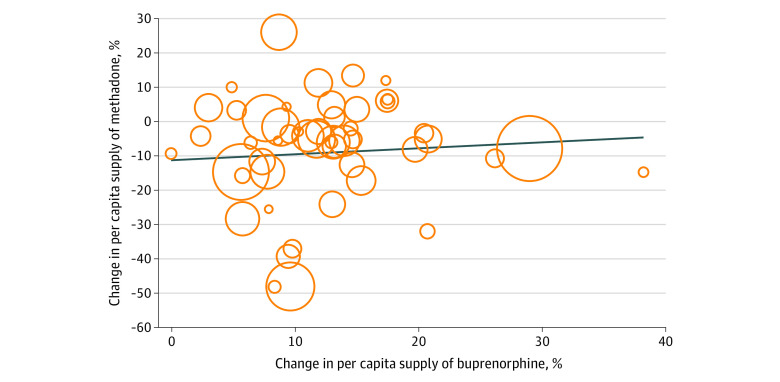
Population-Weighted Scatterplot of Percentage Change in Per Capita Supply of Methadone and Buprenorphine Change in methadone supply was calculated from quarter 3 (Q3) of 2020 to Q2 of 2021 and Q1 to Q4 of 2019; change in buprenorphine supply, from Q3 of 2020 to Q2 of 2021 and Q1 to Q4 of 2019. Results were calculated using ARCOS (Automated Reports and Consolidated Ordering System) data and state population estimates from the US Census Bureau.^27^ The blue line indicates the line of best fit through the scatterplot. The size of a given circle indicates the population weight. Linear fit was 0.17 (95% CI, −0.43 to 0.76; *P* = .47), which indicated no association of increased buprenorphine supply with reduced methadone supply.

In examining the extent to which decreases in the per capita supply of methadone may have been offset by increases in the per capita supply of buprenorphine, we did not observe an association between the 2 growth rates. Figure 3 shows the changes in rates of the per capita methadone supply on the y-axis and in rates of per capita buprenorphine supply on the x-axis during the same period. Although states experiencing increases (relative to other states) in methadone supply experienced larger increases in buprenorphine supply, the linear fit of this association was 0.17 (95% CI, −0.43 to 0.76; *P* = .47), suggesting that increases in buprenorphine supply per capita were not associated with reductions in methadone supply.

## Discussion

Access to medication treatment is crucial for patients with OUD, and the COVID-19 pandemic was associated with disrupted access to care, although the extent and geography of this disruption has been difficult to quantify. Although studies suggest that there has been relatively little disruption to the supply of buprenorphine during the pandemic,^19,20,28,29^ less is known about methadone access during the pandemic. A study examining methadone clinics in the US and Canada found that 1 of 10 clinics were not accepting new patients, and nearly half of those not accepting new patients named the pandemic as the main reason.^30^ A broad expansion of methadone take-home dosing among established patients was found in Washington and Connecticut.^16,31^

Using ARCOS data, we found that although buprenorphine distribution appears relatively unaffected by the pandemic and continues to grow, there was a substantial decline in the methadone supply nationally in the second quarter of 2020, and the supply had not returned to the 2019 level as of quarter 2 of 2021. Pronounced geographic disparities in methadone distribution between states were observed from nearly 50% decreases in New Hampshire and Florida to a 26% increase in Ohio. We found no statistically significant association between increases in the per capita buprenorphine supply and reductions in the per capita methadone supply, suggesting that the reduction in the methadone supply is not offset by supply in buprenorphine at the state level despite the fact that initiation of buprenorphine treatment has fewer requirements than that of methadone. Although more work is certainly needed to understand the implications of this reduced methadone supply, this timely evidence suggests a need for urgent attention to help state and local capabilities treat patients with OUD.

Consistent with past research, we found that the supply of buprenorphine in the US has been increasing since the early 2000s,^15,32^ an increase that continued during the COVID-19 pandemic. This increase is possibly due to legal changes designed to reduce barriers to access medication. For example, one study found an increase in the number of new patients in Texas receiving buprenorphine in the 90 days after the declaration of the COVID-19 public health emergency compared with the 90 days before.^28^ In Pennsylvania, a reduction in rates of both existing patients and new patients initiating buprenorphine treatment were observed; however, there was no overall change in the number of patients with active buprenorphine prescriptions, and the mean days’ supply increased at the same time.^29^ These studies provide a plausible mechanism for the increasing supply of buprenorphine amid the pandemic.

The supply of methadone experienced a sharp reduction at the beginning of the pandemic that persisted for more than 1 year. This outcome is consistent with our hypothesis that barriers to methadone remained notable even with relaxation of restrictions related to MOUD by the Substance Abuse and Mental Health Service Administration. Barriers for access to methadone include the requirement of in-person evaluations for new patients, the requirement to use audiovisual telehealth visits for existing patients, and access to OTPs near patients.^13,14,15^ One study of Medicaid beneficiaries in Wisconsin^33^ found that the COVID-19 public health emergency was associated with decreased probability of urine drug testing and OTP services. Another study of commercially insured patients^21^ found a similar trend of less urine testing across existing and new patients and fewer individuals initiating MOUD. A qualitative study interviewing patients with OUD who had experienced an opioid overdose in the past 3 years^34^ found that adapted substance use disorder treatment policies meant fractures to daily routines for some interviewees. Individuals receiving MOUD have reported having reduced doses or having temporarily stopped receiving MOUD during the pandemic.

Although the literature explaining state disparities in access to methadone is scant, some studies^16,26,33,34^ have suggested that the aforementioned barriers could be exacerbated or mitigated by local governments’ responses to the opioid crisis after the COVID-19 outbreak. States with clear guidance and better funding for OTPs could experience less disruption of methadone supply.^26^ A recent study in Connecticut^16^ showed substantial variability in relaxing methadone treatment requirements among OTPs within the same state, suggesting that more guidance should be provided on the state and federal level to OTPs.

### Limitations

There are limitations to this study. First, distribution is reported in ARCOS by weight rather than prescriptions. We have no information on the number of individuals receiving MOUD with methadone or buprenorphine, about their clinical treatment, and whether they are new patients or were previously receiving MOUD. We also cannot distinguish the use of buprenorphine and methadone to treat pain due to MOUD. However, our analysis of the methadone supply by registrant type using ARCOS data demonstrated that close to 90% of all methadone was distributed at OTPs in 2020 (eFigure 2 in the Supplement), suggesting that declines in methadone used for pain treatment (approximately 10%) are unlikely to be responsible for the decline in supply during the pandemic. Last, this study assessed supply only at the state level. Future research using more granular data is needed to examine within-state variations in per capita supply during the pandemic in the context of prior studies that have documented less access to MOUD in rural areas.^35,36^ Despite these limitations, this work contributes to our understanding of the use of MOUD during the COVID-19 pandemic.

## Conclusions

In this cross-sectional study of buprenorphine and methadone supplies in the US during the COVID-19 pandemic, we observed a stable growth of the buprenorphine supply and a persistent drop in the supply of methadone. The decrease in methadone supply in quarter 2 of 2020 was 20% compared with the previous quarter and 8% compared with the mean level of 2019, and it had not recovered to its 2019 level as of June 2021. These findings suggest that access to buprenorphine was unaffected and access to methadone was disrupted during the pandemic, with great variations across states. Future research examining state disparities is needed to help improve timely access to methadone for patients with OUD.

## Notes

Supplement. **eFigure 1.** Percentage of Buprenorphine Total Supply by Type of Registrant, 2012-2020 **eFigure 2.** Percentage of Methadone Total Supply by Type of Registrant, 2012-2020
