# Supplementary material for: Changes in Buprenorphine and Methadone Supplies in the US During the COVID-19 Pandemic
Source: JAMA Netw Open. 2022 Jul 26;5(7):e2223708. doi: 10.1001/jamanetworkopen.2022.23708 (PMC9327579; doi:10.1001/jamanetworkopen.2022.23708)
Supplement: Supplement. — eFigure 1. Percentage of Buprenorphine Total Supply by Type of Registrant, 2012-2020 eFigure 2. Percentage of Methadone Total Supply by Type of Registrant, 2012-2020 [file jamanetwopen-e2223708-s001.pdf]

Supplementary Online Content

Chen AY, Powell D, Stein BD. Changes in buprenorphine and methadone supplies in the US during the COVID-19 pandemic. *JAMA Netw Open*. 2022;5(7):e2223708. doi:10.1001/jamanetworkopen.2022.23708

eFigure 1. Percentage of Buprenorphine Total Supply by Type of Registrant, 2012-2020

eFigure 2. Percentage of Methadone Total Supply by Type of Registrant, 2012-2020

This supplementary material has been provided by the authors to give readers additional information about their work.

© 2022 Chen AY et al. *JAMA Network Open*.

eFigure 1. Percentage of Buprenorphine Total Supply by Type of Registrant, 2012-2020

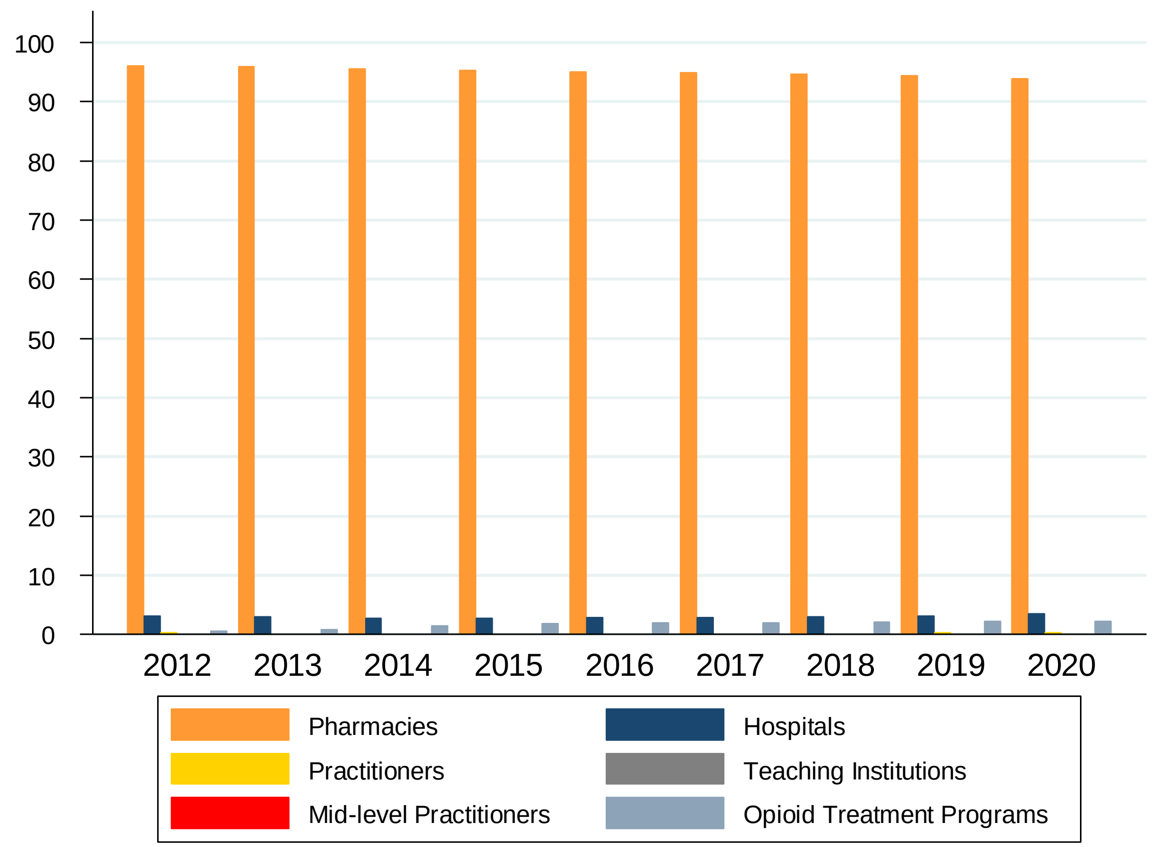

Source/Notes: Authors' calculations using Automated Reports and Consolidated Ordering System (ARCOS) data.

© 2022 Chen AY et al. *JAMA Network Open*.

eFigure 2. Percentage of Methadone Total Supply by Type of Registrant, 2012-2020

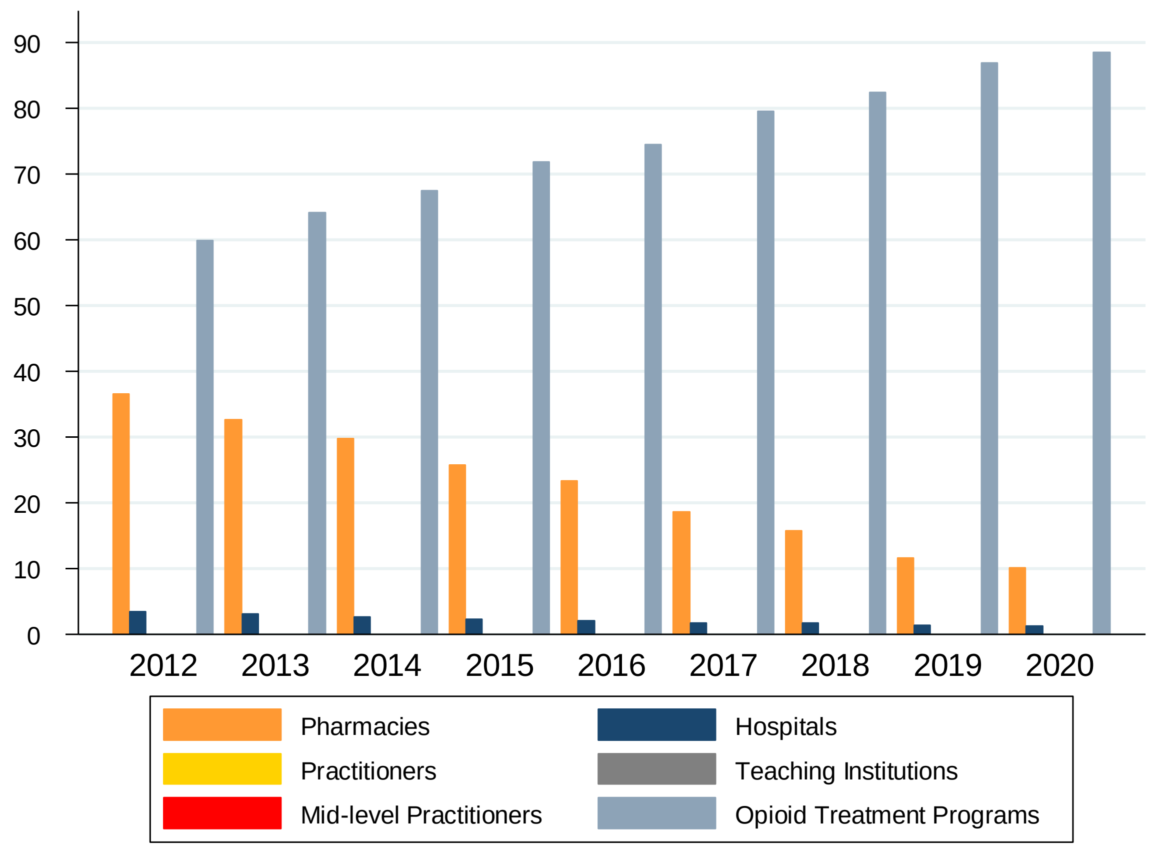

Source/Notes: Authors' calculations using Automated Reports and Consolidated Ordering System (ARCOS) data.

© 2022 Chen AY et al. *JAMA Network Open*.
